# Using Key Predictors in an SVM Model for Differentiating Spinal Fractures and Herniated Intervertebral Discs in Preoperative Anesthesia Evaluation

**DOI:** 10.3390/diagnostics14212456

**Published:** 2024-11-02

**Authors:** Shih-Ying Yang, Shih-Yen Hsu, Yi-Kai Su, Nan-Han Lu, Kuo-Ying Liu, Tai-Been Chen, Kon-Ning Chiu, Yung-Hui Huang, Li-Ren Yeh

**Affiliations:** 1 Department of Anesthesiology, Taoyuan Armed Forces General Hospital, Taoyuan City 30054, Taiwan; ed113786@edah.org.tw; 2 Department of Medical Imaging and Radiological Science, I-Shou University, Kaohsiung City 82445, Taiwan; yhhuang@isu.edu.tw; 3 Department of Information Engineering, I-Shou University, Kaohsiung City 82445, Taiwan; syhsu@isu.edu.tw; 4 Department of Anesthesiology, E-DA Hospital, I-Shou University, Kaohsiung City 82445, Taiwan; jason01cckk@gmail.com; 5 Department of Radiology, E-DA Cancer Hospital, I-Shou University, Kaohsiung City 82445, Taiwan; leunanhan@seed.net.tw (N.-H.L.); ed102500@edah.org.tw (K.-Y.L.); 6 Department of Radiological Technology, Faculty of Medical Technology, Teikyo University, Tokyo 173-8605, Japan; chen.tb@gmail.com; 7 Department of Business Management, National Sun Yat-sen University, Kaohsiung City 82445, Taiwan; chiutony@yahoo.com; 8 Institute of Medical Science and Technology, National Sun Yat-sen University, Kaohsiung City 82445, Taiwan; 9 Department of Anesthesiology, E-DA Cancer Hospital, I-Shou University, Kaohsiung City 82445, Taiwan

**Keywords:** support vector machine, spinal fracture, herniated disc, classification, preoperative anesthesia evaluation

## Abstract

**Background/Objectives:** Spinal conditions, such as fractures and herniated intervertebral discs (HIVDs), are often challenging to diagnose due to overlapping clinical symptoms and the difficulty in assessing their functional impact. Accurate differentiation between these conditions is crucial for effective treatment, particularly in the context of preoperative anesthesia evaluation, where understanding the underlying condition can influence anesthesia planning and pain management. **Methods and Materials:** This study presents a Support Vector Machine (SVM) model designed to distinguish between spinal fractures and HIVDs using key clinical predictors, including age, gender, preoperative Visual Analog Scale (VAS) pain scores, and the number of spinal fractures. A retrospective analysis was conducted on a dataset of 199 patients diagnosed with these conditions. The SVM model, using a radial basis function (RBF) kernel, classified the conditions based on the selected predictors. Model performance was evaluated using precision, recall, accuracy, and the Kappa index, with Leave-One-Out (LOO) cross-validation applied to ensure robust results. **Results:** The SVM model achieved a precision of 92.1% for fracture cases and 91.2% for HIVDs, with recall rates of 98.1% for fractures and 70.5% for HIVDs. The overall accuracy was 92%, and the Kappa index was 0.76, indicating substantial agreement. The analysis revealed that age and VAS pain scores were the most critical predictors for accurately diagnosing these conditions. **Conclusions:** These results highlight the potential of the SVM model with an RBF kernel to reliably differentiate between spinal fractures and HIVDs using routine clinical data. Future work could enhance model performance by incorporating additional clinical parameters relevant to preoperative anesthesia evaluation.

## 1. Introduction

Spinal disorders, such as spinal fractures and herniated intervertebral discs (HIVDs), are widespread conditions that lead to significant disability, impaired mobility, and chronic pain [1,2]. These conditions affect millions of individuals globally and contribute to substantial healthcare costs. Spinal fractures are often associated with trauma, osteoporosis, or degenerative conditions, and their severity can range from mild vertebral compression to more serious fractures requiring surgical intervention [3]. In contrast, an HIVD occurs when the soft inner material of a spinal disc protrudes through a tear in the disc’s outer layer, pressing on nearby nerves and causing pain, numbness, or weakness in the limbs [4,5]. Accurate differentiation between these conditions is particularly important during preoperative anesthesia evaluation, as understanding the underlying diagnosis influences anesthesia planning, pain management, and the overall perioperative strategy.

Diagnosing these conditions accurately is essential for determining appropriate treatment plans. Spinal fractures may require immediate intervention, such as surgery or bracing, to prevent further injury, whereas HIVDs might be managed conservatively with physical therapy or medication unless surgical decompression is necessary. Despite the importance of prompt and accurate diagnosis, distinguishing between spinal fractures and HIVDs can be challenging due to overlapping symptoms, such as back pain, leg pain, and neurological impairments. Medical imaging techniques, such as magnetic resonance imaging (MRI) and computed tomography (CT) scans [6], combined with clinical evaluations, are standard diagnostic tools. However, these techniques may not always provide definitive answers due to the subtlety of the differences between these conditions [7,8]. In recent years, machine learning methods have been employed to aid in medical diagnostics, offering a potential solution to this diagnostic challenge [9,10]. This study seeks to leverage Support Vector Machine (SVM) models to enhance the accuracy of diagnosing spinal fractures and HIVDs based on clinical predictors.

## 2. Literature Review

Key clinical predictors play a vital role in distinguishing between spinal fractures and HIVDs. While imaging is a cornerstone of diagnostic work, it is often complemented by patient-specific data such as age, gender, and the severity of pain [11,12,13,14]. For instance, age is a significant risk factor, as spinal fractures are more common in older individuals, particularly those with osteoporosis, whereas an HIVD is typically seen in middle-aged adults. Gender differences also influence the prevalence of these conditions, with men generally at higher risk for spinal fractures and HIVDs due to different patterns of bone density loss and occupational hazards [15,16]. The Visual Analog Scale (VAS) for pain is another crucial predictor, offering a quantitative measure of a patient’s pain experience [17,18,19]. Pain intensity can help differentiate between the conditions, with certain patterns of pain correlating more strongly with fractures than with HIVDs [20]. Additionally, the number of spinal fractures can serve as an indicator of the severity and complexity of a spinal condition, further refining the diagnostic process. These clinical features, when considered together, provide valuable information that can improve diagnostic accuracy. Utilizing them in a machine learning model, such as an SVM, can help distinguish between spinal fractures and HIVDs, leading to more tailored treatment strategies. This study leverages these predictors to create a model that assists in differentiating these conditions, ultimately improving diagnostic precision.

Despite the availability of advanced imaging techniques and clinical tools, classifying spinal fractures and HIVDs remains a challenging task for several reasons. First, the symptoms of both conditions often overlap, leading to potential misdiagnoses. Back pain, radicular pain, and even neurological deficits are common in both conditions, and clinical presentations can be subtle or vary significantly among patients. Even with high-resolution MRI or CT scans, fractures and disc herniations may be difficult to differentiate, particularly when they occur concurrently or in patients with complex medical histories [21,22,23,24,25]. Another challenge arises from the variability in the severity and location of spinal conditions. For example, minor fractures may not produce obvious symptoms, while severe HIVD cases can mimic the clinical presentation of fractures. Furthermore, inter-individual differences, such as varying pain tolerances and the presence of comorbidities, add complexity to the classification process. These diagnostic ambiguities can result in delays in appropriate treatment, incorrect interventions, or prolonged suffering for patients. Traditional diagnostic methods heavily rely on the subjective judgment of radiologists and clinicians, which can lead to variability in diagnosis and treatment planning. This subjectivity, combined with the complexity of spinal anatomy and overlapping clinical symptoms, underscores the need for a more objective, data-driven approach to classification. Machine learning models, such as SVMs, offer a promising solution by leveraging clinical data to identify subtle patterns and enhance diagnostic accuracy.

Support Vector Machines (SVM) have emerged as a powerful tool in medical classification tasks due to their ability to handle high-dimensional and complex data. SVM is a supervised machine learning algorithm that excels in binary classification problems, making it ideal for distinguishing between two conditions, such as spinal fractures and HIVDs. The core principle of SVM is to find the optimal hyperplane that maximizes the margin between two classes. In the context of this study, the two classes are patients diagnosed with spinal fractures and those diagnosed with HIVDs. By maximizing the margin between these classes, SVM can achieve better generalization and perform well on new, unseen data. Furthermore, SVM models are less prone to overfitting, especially when working with small- to medium-sized datasets, which is a common scenario in medical research. This is particularly important when the dataset contains only a limited number of cases, such as the 199 patients included in this study. The robustness of SVM in handling small datasets makes it an attractive choice for medical applications where the availability of labeled data is often a limiting factor. SVM is chosen for its strengths in handling nonlinear and complex data, its robustness with smaller datasets, and its successful track record in medical applications. The algorithm’s ability to integrate diverse clinical predictors into a cohesive model makes it a powerful tool for enhancing diagnostic accuracy in cases where traditional methods may fall short.

## 3. Materials and Methods

### 3.1. Ethics Approval

This study was conducted in accordance with the ethical standards outlined in the Declaration of Helsinki and approved by the institutional review board (IRB) at the E-AD Hospital Taiwan with approval No. EDCHP113013. Since this research involved retrospective data collection from medical records and imaging studies, informed consent was waived by the IRB. All patient data were anonymized to protect confidentiality. No direct patient interaction occurred during the study, and the data was used exclusively for research purposes. The ethics approval ensured that patient privacy and data protection measures were rigorously maintained throughout the study.

### 3.2. Diagram of Research

**Figure 1:**
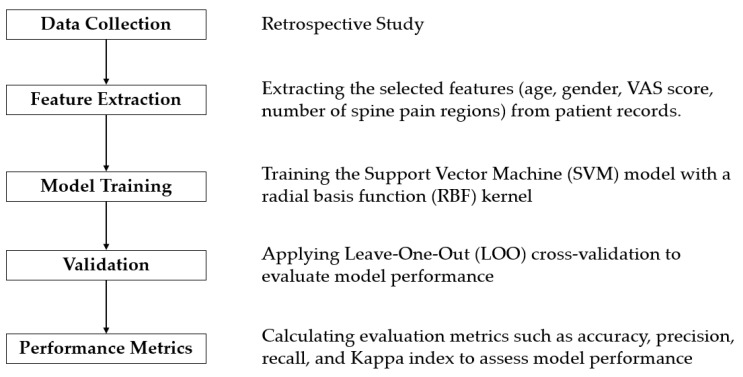
Diagram of research.

The research diagram is presented in Figure 1, summarizing the main steps of the proposed method. First, clinical data, including age, gender, VAS pain scores, number of spine pain regions, and MRI reports, were collected from 199 patients diagnosed with either spinal fractures or HIVDs. Next, the selected features (age, gender, VAS score, number of spine pain regions) were extracted from patient records. The Support Vector Machine (SVM) model, with a radial basis function (RBF) kernel, was then trained using the collected data. To evaluate the model’s performance, Leave-One-Out (LOO) cross-validation was applied. Finally, evaluation metrics such as accuracy, precision, recall, and the Kappa index were calculated to assess the model’s effectiveness.

### 3.3. Data

A retrospective analysis was performed on a cohort of 199 individuals who were diagnosed with spinal conditions, specifically spinal fractures (*n* = 155) and herniated intervertebral discs (HIVDs) (*n* = 44). The patients included in the study had been diagnosed based on magnetic resonance imaging (MRI) of the spine, which is considered the gold standard for evaluating both spine fractures and disc herniation. The data were collected from patient medical records, including demographic information and clinical assessments conducted prior to any surgical interventions.

The inclusion criteria were: (1) diagnosis of either a spinal fracture or HIVD, confirmed by MRI imaging, and (2) availability of preoperative clinical data, including Visual Analog Scale (VAS) scores and the number of spinal pain regions assessed through the MRI report. Patients with incomplete data or unclear MRI diagnoses were excluded from the study. The final dataset included 155 cases of spinal fractures and 44 cases of HIVD.

### 3.4. Key Predictors

Key clinical predictors were selected based on their relevance to differentiating between spinal fractures and HIVDs. These predictors are commonly available in routine clinical practice and have been shown to be associated with spinal conditions. The predictors used for classification in the SVM model are as follows.

Age: Patients’ age at the time of diagnosis was included as a continuous variable. Age is a crucial factor, as spinal fractures are more prevalent in older populations, particularly those with osteoporosis, whereas HIVDs tend to affect middle-aged adults.

Gender: Gender was recorded as a binary variable (male or female). Gender has been shown to influence the prevalence and severity of both spinal fractures and HIVD, with males being more prone to spinal fractures.

VAS Score Before Surgery: The Visual Analog Scale (VAS) was used to quantify the intensity of pain experienced by the patients before any surgical interventions. The VAS is a 0–10 scale, where 0 represents no pain and 10 represents the worst possible pain. VAS scores provide an objective measure of the patient’s perceived pain level and help distinguish between the severity of spinal fractures and HIVDs.

**Figure 2:**
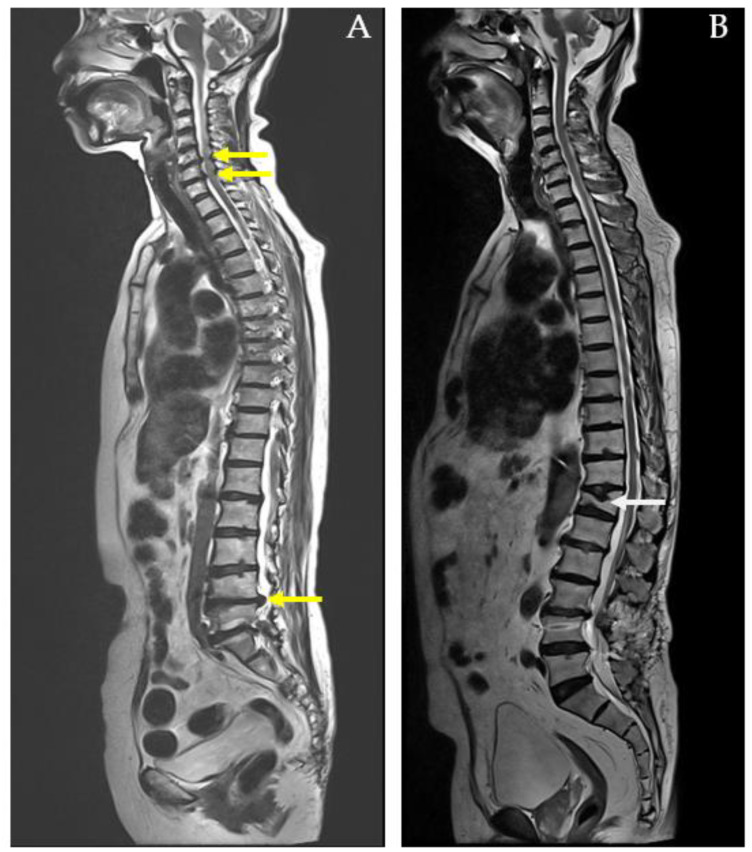
MRI whole-spine imaging demonstrating herniated intervertebral discs (HIVDs) indicated by yellow arrow in (**A**) and compression fracture indicated by white arrow in (**B**).

Number of Spine Pain Regions: The number of painful regions in the spine was assessed using the MRI report, which offers an objective measure of pain distribution and severity. This clinical examination identifies areas of tenderness and helps differentiate between conditions involving localized versus widespread spinal involvement. For example, a 73-year-old female with herniated intervertebral discs (HIVDs) at the C5-C6-C7 and L4-L5-S1 levels had six identified spine pain regions (Figure 2A). In contrast, a 77-year-old male with a compression fracture at T12 had only one identified spine pain region (Figure 2B).

Diagnosis: The two diagnostic categories, spinal fracture (*n* = 155) and HIVD (*n* = 44), were established based on MRI imaging of the spine (Figure 1). This binary classification was used as the target variable for the SVM model, with the diagnostic outcomes serving as the response variable.

#### The Process of Feature Extraction and Selection

(I) Clinical Predictors Selection: The clinical predictors used in this study—age, gender, preoperative Visual Analog Scale (VAS) pain scores, and the number of spinal pain regions—were selected based on their clinical relevance and established association with spinal conditions. The selection was informed by the previous literature, which indicated that these features are significant indicators for distinguishing between spinal fractures and HIVDs. Specifically:
  ➢ Age: Spinal fractures are more common in older adults, particularly those with osteoporosis, while HIVDs are more prevalent among middle-aged adults.
  ➢ Gender: Gender differences can influence the prevalence of both conditions, with males generally showing higher rates of fractures and HIVDs.
  ➢ VAS Pain Score: The severity of pain, as quantified by the VAS, provides an important distinction, as certain pain patterns are more characteristic of one condition than the other.
  ➢ Number of Spine Pain Regions: This feature was included to assess the distribution of pain, which could help differentiate between localized spine fractures and more widespread disc herniation.
(II) Feature Extraction: The selected features were extracted from patient records, including MRI reports and clinical evaluations conducted before any surgical interventions. The data for each patient were collected retrospectively, and features were obtained as follows:
  ➢ Age and Gender: Directly recorded from patient demographic information.
  ➢ VAS Score: Extracted from preoperative assessments, where each patient rated their pain on a scale from 0 (no pain) to 10 (worst possible pain).
  ➢ Number of Spine Pain Regions: Determined by reviewing MRI reports, which provided information on the locations of pain, as well as clinical notes regarding the patient’s physical examination.
(III) Feature Selection Process: To ensure that the features used in the Support Vector Machine (SVM) model contributed meaningfully to the classification task, a correlation analysis was conducted to examine relationships among predictors. Features with high correlation were carefully reviewed to avoid redundancy and ensure the stability of the model. Pearson correlation coefficients indicated that age and VAS pain scores were the most significant predictors, while the number of spine pain regions and the patient’s gender showed minimal impact on model performance. This informed the decision to keep all features in the final model, as they were found to add value in differentiating the two conditions.

### 3.5. SVM Model

A Support Vector Machine (SVM) model was employed to classify spinal fractures and HIVDs based on clinical predictors. SVM is a supervised learning algorithm that is widely used for classification tasks, particularly when the dataset is relatively small and the features are complex. The SVM model works by finding the optimal hyperplane that maximally separates the data points of the two classes—in this case, spinal fractures and HIVDs. Given the predictors *x*_1_, *x*_2_, *x*_3_, *x*_4_ (age, gender, VAS before surgery, number of spine pain regions), and the response Y (diagnosis: 1 for spinal fracture, 0 for HIVD), the SVM model seeks to find the optimal hyperplane that separates the two classes.

For this study, an SVM with a radial basis function (RBF) kernel was used. The RBF kernel is effective for handling nonlinear relationships between the input variables and the output classes, making it suitable for medical data where clinical variables may not have simple linear relationships. The RBF kernel transforms the input data into a higher-dimensional space, allowing the model to create a more accurate decision boundary between the two classes. The parameters of the SVM model, including the penalty parameter (C) and the kernel width (γ), were optimized through cross-validation to ensure the best possible classification performance (Table 1).

### 3.6. Evaluation of Classification

To evaluate the performance of the SVM model, several standard classification metrics were used: precision, recall, accuracy, and the Kappa index. We defined TP as True Positive, TN as True Negative, FP as False Positive, and FN as False Negative.

Precision refers to the proportion of correctly predicted positive instances (true positives) out of all instances predicted as positive as Equation (1). *Precision* was calculated for both the fracture and HIVD classifications to assess the model’s ability to correctly identify each condition.

(1) Precision=TP/(TP+FP)

*Recall*, also known as sensitivity, measures the proportion of actual positive instances that were correctly identified by the model as Equation (2). This metric was used to evaluate how well the model identified true cases of spinal fractures and HIVDs.

(2) Recall=TP/(TP+FN)

*Accuracy* is the proportion of correct predictions (both true positives and true negatives) out of the total number of cases as Equation (3). It provides a general measure of the model’s performance across both classes.

(3) Accuracy=(TP+TN)/(TP+TN+FP+FN)

The *Kappa* index is a measure of inter-rater agreement that accounts for chance. It was used in this study to assess the agreement between the model’s predictions and the actual diagnoses, where *P_o_* is the observed agreement and *P_e_* is the expected agreement by chance as Equation (4). The Kappa index helps understand the model’s performance beyond simple accuracy, especially in cases of imbalanced classes.

(4) $$Kappa=(P_{o}-P_{e})/(1-P_{e})$$

The *Kappa* index further measures the agreement between predicted and actual classifications, accounting for chance, which is critical in the context of imbalanced data. A higher Kappa value indicates greater agreement between the model and the true classification.

To validate the model, Leave-One-Out (LOO) cross-validation was employed. LOO is a form of cross-validation where one observation is left out in each iteration as the test set, while the remaining data are used for training. This process is repeated for every observation in the dataset, and the average performance across all iterations is reported. LOO cross-validation is particularly suitable for small datasets, as it maximizes the use of available data and reduces the risk of overfitting. This approach also helped us evaluate how well the model generalizes to new, unseen data and minimizes the risk of overfitting. Meanwhile, Pearson correlation coefficient analysis was conducted to examine the collinearity among the predictors. Receiver Operating Characteristic (ROC) analysis was used to evaluate the performance of the SVM model.

## 4. Results

### 4.1. Descriptive Statistics of Key Clinical Predictors

Table 2 presents the descriptive statistics of key clinical predictors used in the classification of spinal fractures (*n* = 155) and herniated intervertebral discs (HIVDs) (*n* = 44) cases. The variables include VAS Score Before Surgery, Age, and Number of Spine Pain Regions, with corresponding mean, median, standard deviation (STD), minimum (Min), and maximum (Max) values for each diagnosis group. Patients with spinal fractures had an average VAS score of 6.2, while HIVD patients had a higher average score of 7.4. The median scores were 6.0 and 8.0 for the fracture and HIVD groups, respectively. The standard deviation was 1.5 for the fracture group and 1.3 for the HIVD group. The mean age of fracture patients was significantly higher at 73.0 years compared to 49.0 years for HIVD patients. The age range for fracture patients spanned from 41 to 92 years, while for HIVD patients, it ranged from 16 to 88 years. Fracture patients had an average of 1.5 spine pain regions, with a median of 1.0, while HIVD patients had a higher average of 2.1 regions, with a median of 2.0. The standard deviation was 0.9 for fractures and 0.3 for HIVDs.

Table 3 provides descriptive statistics of the key clinical predictors—VAS Score Before Surgery, Age, and Number of Spine Pain Regions—broken down by gender. Female patients had a mean VAS score of 6.4, with a median of 6.0, while male patients had a slightly higher mean score of 6.7 and a median of 7.0. The standard deviations were 1.6 and 1.5 for females and males, respectively. The mean age of female patients was 71.0 years, with a median of 73.0 years, compared to 59.0 years for male patients, who had a median of 65.0 years. The age range for females was from 38 to 90 years, while for males, it ranged from 16 to 92 years. Female patients had an average of 1.6 spine pain regions, with a median of 1.0, while male patients had a mean of 1.8 and a median of 2.0. The standard deviations were 0.8 for females and 0.9 for males, with both genders having a minimum of 1.0 and a maximum of 6.0 pain regions.

### 4.2. Correlation Among Key Predictors

Table 4 presents the Pearson correlation coefficients between three variables—VAS, Age, and the Number of Spine Pain Regions—separately for female and male patients. Significant correlations are marked with *p* < 0.01 and *p* < 0.05. For female patients, there were no significant correlations observed between VAS, Age, and the Number of Pain Regions, with correlation coefficients all being near zero. For male patients, significant negative correlations were observed between VAS and Age (r = −0.554, *p* < 0.01), and between Age and the Number of Spine Pain Regions (r = −0.253, *p* < 0.05), indicating that older males tended to report lower VAS pain scores and fewer pain regions. No significant correlation was found between VAS and the Number of Spine Pain Regions in males (r = 0.110).

When significant correlations exist between predictors (also known as multicollinearity) in a machine learning model like the Support Vector Machine (SVM), it can have several effects on the model’s performance. If two or more predictors are highly correlated, they provide similar information to the SVM model. This redundancy can lead to inefficiencies in the learning process because the model may give undue importance to these redundant predictors. Meanwhile, predictors are highly correlated, and small changes in the data can lead to significant shifts in the model’s decision boundary. This makes the model less stable and less able to generalize well to new, unseen data. While SVM is robust to some degree of multicollinearity, significant correlations between predictors can still impact the model’s stability, accuracy, and interpretability. Addressing multicollinearity through feature selection, dimensionality reduction, or regularization can improve the performance and generalizability of the SVM model.

### 4.3. Classification by SVM

Table 5 shows the classification performance of the SVM model when different combinations of key predictors (VAS, Gender, Age, Number of Spine Pain Regions) are included or excluded. The table provides metrics such as True Class (Fracture/HIVD), Precision, Recall, False Positives (FP), False Negatives (FN), Accuracy, and Kappa index for each scenario.

(A) Predicted Class with All Key Predictors: Fracture cases were predicted with a recall of 98.06%, and HIVDs with a recall of 70.45%. Precision for Fracture was 92.12%, and for HIVD, 91.18%. Overall accuracy was 92%, with a Kappa value of 0.75.
(B) Without VAS: Excluding VAS led to a drop in performance: recall for HIVD dropped to 63.64%, and precision for HIVD dropped to 87.50%. Overall accuracy fell to 90%, and Kappa dropped to 0.69, indicating a substantial impact on the model’s ability to classify HIVD accurately.
(C) Without Gender: Excluding gender did not change the performance from the full predictor model. Recall, Precision, Accuracy (92%), and Kappa (0.75) were identical to the full model, suggesting that gender did not significantly affect classification outcomes.
(D) Without Age: Excluding Age resulted in a notable decline in performance. The recall for HIVD dropped to 47.73%, and the precision for HIVD fell to 65.63%. The overall accuracy dropped to 83%, and Kappa decreased significantly to 0.47, highlighting the importance of Age as a predictor.
(E) Without Number of Spine Pain Regions: Excluding the Number of Spine Pain Regions had no impact on the model’s performance compared to the full model, maintaining a 92% accuracy and a Kappa value of 0.75, indicating that this variable may not significantly contribute to classification accuracy.
(F) Without Gender and Number of Spine Pain Regions: Excluding both Gender and Number of Spine Pain Regions also maintained the same performance as the full model, with an accuracy of 92% and a Kappa value of 0.75.

Age and VAS are crucial predictors for accurate classification, as their removal substantially reduces the model’s performance, particularly for HIVD cases. Gender and Number of Spine Pain Regions appear to have little impact on the model’s classification performance, as their exclusion did not affect the results.

**Figure 3:**
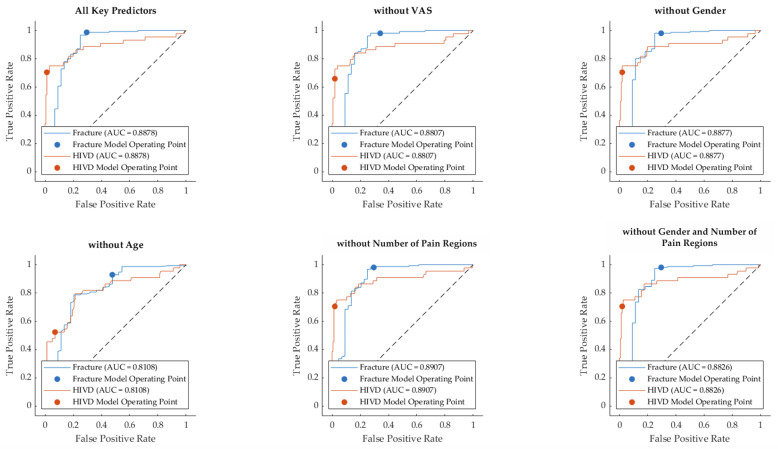
Receiver operating characteristic (ROC) curves for SVM models with different combinations of key predictors.

Figure 3 displays the receiver operating characteristic (ROC) curves for the SVM model’s classification of spinal fractures and HIVDs across six different scenarios: with all key predictors and with certain predictors excluded. Each plot shows the true positive rate (sensitivity) against the false positive rate (1-specificity) for both spinal fractures and HIVDs. The area under the curve (AUC) values for each model are presented to assess the performance of the classifier.

The AUC for both fracture and HIVD classification was 0.8878, indicating strong model performance with all predictors included. Excluding VAS reduced the AUC slightly to 0.8807 for both fractures and HIVD. This suggests that while the model’s performance slightly declined, it still maintained relatively high discriminative power. The AUC values remained unchanged at 0.8877, indicating that removing gender had no impact on the model’s performance. Excluding age resulted in a notable drop in performance, with the AUC decreasing to 0.8108 for both conditions. This suggests that age is a crucial predictor for distinguishing between spinal fractures and HIVDs. Removing the number of spine pain regions did not negatively impact the model, as the AUC increased slightly to 0.8907, indicating that this variable did not contribute significantly to the model’s classification ability. The AUC for both fracture and HIVD classification remained high at 0.8826, suggesting that the exclusion of both gender and the number of pain regions did not considerably affect model performance. The inclusion of Age and VAS as predictors contributed significantly to the model’s performance, while Gender and Number of Pain Regions had minimal impact on the classification results. The model performed well in all scenarios, with AUC values consistently high, but the exclusion of Age caused the largest drop in performance.

## 5. Discussion

The primary motivation for adopting the Support Vector Machine (SVM) rather than deep learning lies in the nature of the dataset and the practical aspects of its application. Specifically, the dataset used in our study comprises 199 patient records, which is relatively small for training deep learning models effectively. Deep learning models typically require a large volume of data to avoid overfitting and to achieve optimal performance. On the other hand, the SVM is well suited for small- to medium-sized datasets, where it can achieve good generalization without requiring the extensive computational resources and training data that deep learning models demand. One of our objectives was to create a model that could provide interpretable insights into the role of key clinical predictors such as age, gender, and VAS pain scores. Traditional machine learning methods like SVMs are more interpretable compared to deep learning models, which are often viewed as “black boxes.” Clinicians are more inclined to use models that offer transparent reasoning for their predictions, especially in preoperative anesthesia evaluation, where understanding the importance of clinical features is crucial for decision-making. Deep learning models are computationally intensive, requiring significant processing power and specialized hardware such as GPUs. By contrast, SVMs can be trained efficiently on standard hardware, making them more accessible for real-time clinical applications, particularly in resource-limited settings. This study aims to develop a practical tool for differentiating between spinal fractures and HIVDs using readily available clinical data. Considering the dataset size and the desire for an interpretable and efficient model, the SVM was a suitable choice that aligns well with our practical and clinical goals.

This study demonstrates the utility of an SVM model in differentiating between spinal fractures and HIVDs using key clinical predictors such as age, gender, VAS pain scores, and the number of spine pain regions. The findings highlight several critical insights into the predictive value of these variables, as well as the strengths and limitations of machine learning approaches in the clinical diagnosis of spinal conditions.

### 5.1. Importance of Key Predictors

Among the key predictors included in the model, age and VAS scores were shown to be the most influential in predicting the correct diagnosis of spinal fracture or HIVD. The exclusion of age (Table 4, panel D) resulted in a significant decrease in the model’s performance, as reflected by the drop in both accuracy (83%) and the area under the curve (AUC) (0.8108). This finding aligns with the clinical understanding that age is a critical factor in the etiology of spinal conditions. Spinal fractures, particularly those related to osteoporosis, are more common in older populations, while HIVDs tend to occur more frequently in younger adults. Thus, the strong dependence of the model on age underscores its importance in distinguishing between these conditions. Similarly, the exclusion of VAS pain scores led to a drop in both precision and recall for HIVD classification, resulting in a lower overall AUC (0.8807). Pain intensity, as measured by the VAS, plays a significant role in clinical assessments, and the model’s reliance on this variable demonstrates its importance in diagnosis. HIVDs often present with more severe radiating pain compared to fractures, which may explain why the model struggled to classify HIVD cases accurately without the VAS scores.

On the other hand, the predictors gender and number of spine pain regions had minimal impact on the model’s performance. The exclusion of these variables did not result in any significant drop in accuracy or AUC, suggesting that these factors may not be as crucial for differentiating between spinal fractures and HIVDs. This finding is consistent with prior research, which shows that while gender may influence the prevalence of certain spinal conditions, it is not a strong diagnostic predictor on its own.

Table 6 summarizes various classification methods used in spine-related research, comparing their precision and accuracy. The presented method, which uses an SVM with key predictors, achieves the highest precision (0.981) among the compared methods, indicating its effectiveness in distinguishing between HIVDs and spinal fractures.

### 5.2. Impact of Multicollinearity and Model Robustness

The presence of significant correlations between the predictors, as shown in the Pearson correlation analysis, raised concerns about multicollinearity and its potential effects on the SVM model. However, the SVM model appeared robust to the effects of multicollinearity, as removing correlated predictors like gender and number of pain regions did not significantly affect model performance. This indicates that the SVM algorithm effectively managed redundancy in the data, possibly due to its margin-maximizing properties and ability to handle complex relationships between variables through kernel functions, such as the radial basis function (RBF) used in this study.

Nonetheless, the importance of age and VAS suggests that even in the presence of multicollinearity, certain predictors carry more diagnostic weight. It may be beneficial to consider dimensionality reduction techniques like principal component analysis (PCA) or regularization techniques in future studies to address multicollinearity explicitly and further optimize the model.

### 5.3. Clinical Implications

The high performance of the SVM model, particularly when all key predictors were included (accuracy of 92%, AUC of 0.8878), highlights its potential as a diagnostic tool in clinical settings. The ability of the model to accurately classify cases based on routine clinical data, including age and pain scores, could aid clinicians in making more informed diagnostic decisions, especially when imaging results are ambiguous or delayed. This is particularly relevant for rural or resource-limited settings where access to advanced imaging modalities like MRI may be restricted. However, the findings also suggest that the exclusion of key clinical variables, particularly age and VAS scores, can significantly impair the model’s diagnostic performance. Therefore, it is critical that clinicians ensure comprehensive data collection to optimize the effectiveness of such machine learning tools.

Spinal conditions such as fractures and HIVDs are common and can have overlapping clinical symptoms, making accurate diagnosis challenging. Misdiagnosis or delayed diagnosis can lead to inappropriate treatments, which may result in prolonged pain, unnecessary procedures, or worsening of the condition. By developing a machine learning model like the SVM, this study contributes to improving diagnostic accuracy. Accurate differentiation between spinal fractures and HIVDs is critical to ensuring that patients receive the most appropriate and timely treatment.

In many cases, MRI is used to confirm diagnoses of spinal conditions. However, access to advanced imaging techniques like MRI may be limited in resource-constrained settings or rural areas. This research shows that routine clinical data (age, gender, pain scores, number of spine pain regions) can be used to build a predictive model, reducing the reliance on expensive and less accessible diagnostic tools. This can be particularly valuable for healthcare providers who lack immediate access to advanced imaging technology. The integration of machine learning models into clinical practice has the potential to assist clinicians in making more informed, data-driven decisions. In complex cases where symptoms overlap or clinical uncertainty exists, having an additional diagnostic tool can aid healthcare providers in confirming or refining their diagnoses. This SVM model offers clinicians a supplementary tool to enhance the diagnostic process, potentially improving patient outcomes through better-informed decision-making. Machine learning models like the one developed in this study can process large amounts of data quickly, providing diagnostic insights more rapidly than traditional methods that rely on clinician interpretation of imaging or physical examinations. This increase in diagnostic efficiency could be particularly important in busy healthcare environments where timely decision-making is crucial for patient management.

**Figure 4:**
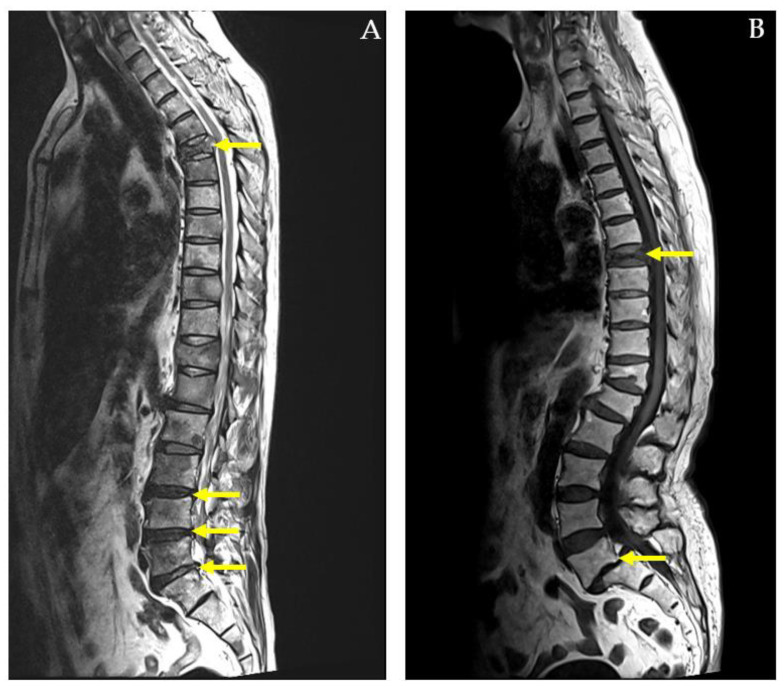
MRI whole-spine imaging demonstrating pathological compression fractures, herniated intervertebral discs (HIVDs), and spondylolisthesis indicated by yellow arrows. In (**A**) a 59-year-old male diagnosed with lung cancer and diffuse bony metastases affecting the cervical, thoracic, lumbar spine, sacrum, bilateral iliac bones, and right femoral head. The MRI reveals a pathological compression fracture of the T5 vertebral body with associated spinal cord compression. Additionally, herniated intervertebral discs (HIVDs) are present at the L3-L4, L4-L5, and L5-S1 levels. In (**B**) an 87-year-old female with a compression fracture at the T8 vertebral body. The MRI also shows L5-S1 spondylolytic spondylolisthesis with narrowing of bilateral neural foramina.

This research is important because it provides a pathway to more accurate, efficient, and cost-effective diagnostics for spinal conditions, enhances clinical decision-making, and contributes to the broader integration of machine learning into personalized healthcare. Furthermore, the study did not account for complex cases, such as those depicted in Figure 4, where patients presented with both pathological compression fractures and diffuse bony metastases, or with multiple spinal conditions like spondylolytic spondylolisthesis.

## 6. Conclusions

This study demonstrates the effectiveness of an SVM model in distinguishing between spinal fractures and HIVDs using key clinical predictors such as age, gender, VAS pain scores, and the number of spine pain regions. The results show that age and VAS pain scores are crucial predictors for accurate classification, while gender and the number of spine pain regions have less influence on the model’s performance. The model achieved high accuracy (92%) and a substantial agreement, as indicated by a Kappa value of 0.75, suggesting that the SVM is a reliable tool for diagnosing these conditions based on routine clinical data. One of the significant findings is that excluding age and VAS from the model drastically reduces its accuracy, highlighting the importance of these variables in clinical diagnosis. The use of machine learning, particularly the SVM with an RBF kernel, proves to be a valuable approach for analyzing complex medical datasets, where clinical symptoms and characteristics overlap. Additionally, the model’s robustness against multicollinearity suggests its potential to handle clinical data with correlated predictors.

The practical implications of this research are promising. The SVM model offers clinicians a supplementary diagnostic tool that can improve the accuracy of diagnosis, reduce reliance on advanced imaging techniques, and potentially lower healthcare costs. By using readily available clinical data, this model can aid in decision-making, especially in resource-limited settings where access to advanced imaging is restricted. However, the study also identifies areas for further research. Future studies should focus on expanding the dataset, incorporating additional clinical parameters, and exploring alternative machine learning algorithms to enhance the generalizability and performance of the model. Moreover, improving the interpretability of machine learning models in clinical practice will be crucial for increasing their adoption and trust among healthcare professionals.

This study provides a strong foundation for the integration of machine learning into the diagnosis of spinal conditions. The SVM model developed here demonstrates high diagnostic potential, offering a step forward in the application of artificial intelligence to improve clinical outcomes and personalized care in patients with spinal fractures and HIVDs.

## 7. Limitations and Future Directions

While this study demonstrates promising results, several limitations should be noted. First, the retrospective nature of the dataset introduces potential biases related to data collection and diagnostic accuracy. Future studies could address this limitation by conducting prospective analyses with standardized data collection protocols. Additionally, the dataset included a limited number of HIVD cases (*n* = 44), which may have impacted the model’s ability to generalize to larger and more diverse populations. Expanding the dataset to include more cases of both spinal fractures and HIVDs would improve the model’s generalizability and robustness. Moreover, while the SVM model performed well, other machine learning algorithms, such as deep learning models or ensemble methods, could be explored in future studies to determine if they offer any advantages in predictive accuracy or interpretability. Integrating additional clinical parameters, such as patient comorbidities or more detailed imaging features, could also enhance model performance.

While the SVM model provides useful classification results, its lack of interpretability in comparison to simpler models like logistic regression may limit its clinical utility. Clinicians may require more transparent models that provide clear explanations for their predictions. Thus, future work could focus on developing interpretable machine learning models that offer high accuracy without sacrificing transparency. Furthermore, the study did not account for complex cases, where patients presented with both pathological compression fractures and diffuse bony metastases, or with multiple spinal conditions like spondylolytic spondylolisthesis. These more complicated cases were not fully addressed in the model, which could limit its application in more severe or multifactorial clinical scenarios. Future studies should incorporate these complex cases to improve the model’s comprehensiveness and clinical relevance.

## Figures and Tables

**Table 1:** SVM parameter configuration for classification of spinal fractures and HIVDs.

Parameter	Description	Value/Configuration
Regularization (C)	Controls the trade-off between maximizing the margin and minimizing classification errors. A larger value penalizes misclassifications more heavily.	Tuned via cross-validation
Kernel	Defines the kernel function used by the SVM to transform the data into a higher dimension. RBF kernel was used for capturing nonlinear relationships.	Radial basis function (RBF)
Gamma (γ)	Determines how far the influence of a single training example reaches, affecting the decision boundary’s shape.	Optimized via grid search
Degree (Polynomial)	Specifies the degree of the polynomial kernel (only applicable for polynomial kernel).	Not applicable (RBF used)
Class Weight	Adjusts weights to handle class imbalance, assigning higher importance to the minority class to reduce bias.	Balanced
Max Iterations	Sets the maximum number of iterations allowed for the optimization algorithm to converge.	10,000
Tolerance (ε)	Defines the stopping criterion for the optimization process, balancing precision and computational effort.	0.001

**Table 2:** Descriptive statistics of key clinical predictors for spinal fracture and HIVD groups.

Variable	Diagnosis	*n*	Mean	Median	STD	Min	Max
VAS Score Before Surgery	Fracture	155	6.2	6.0	1.5	3.0	9.0
	HIVD	44	7.4	8.0	1.3	3.0	8.0
Age	Fracture	155	73.0	74.0	9.1	41.0	92.0
	HIVD	44	49.0	45.5	16.4	16.0	88.0
Number of Spine Pain Regions	Fracture	155	1.5	1.0	0.9	1.0	6.0
	HIVD	44	2.1	2.0	0.3	1.0	3.0

**Table 3:** Descriptive statistics of key clinical predictors by gender.

Variable	Gender	*n*	Mean	Median	STD	Min	Max
VAS Score Before Surgery	Female	144	6.4	6.0	1.6	3.0	9.0
	Male	55	6.7	7.0	1.5	3.0	8.0
Age	Female	144	71.0	73.0	11.3	38.0	90.0
	Male	55	59.0	65.0	19.3	16.0	92.0
Number of Spine Pain Regions	Female	144	1.6	1.0	0.8	1.0	6.0
	Male	55	1.8	2.0	0.9	1.0	6.0

**Table 4:** Pearson correlation coefficients among VAS, age, and number of spine pain regions by gender.

Gender	Variable	VAS	Age	Number of Pain Regions
Female	VAS	1.000	−0.053	0.058
	Age	−0.053	1.000	−0.116
	Number of Pain Regions	0.058	−0.116	1.000
Male	VAS	1.000	−0.554 **	0.110
	Age	−0.554 **	1.000	−0.253 *
	Number of Pain Regions	0.110	−0.253 *	1.000

** *p* < 0.01 and * *p* < 0.05.

**Table 5:** Classification results of SVM models with different sets of key predictors for spinal fracture and HIVD diagnosis. Note: Precision, Recall, False Positives (FP), and False Negatives (FN) are expressed as percentages (%), while Accuracy and Kappa values are expressed as values between 0 and 1.

**(A) Predicted Class with all Key Predictors**							**(B) without VAS**					
**True Class**	**Fracture**	**HIVD**		**Recall**	**FP**		**True Class**	**Fracture**	**HIVD**		**Recall**	**FP**
**Fracture**	**152**	3		**98.06**	1.94		**Fracture**	**151**	4		**97.42**	2.58
**HIVD**	13	**31**		**70.45**	29.55		**HIVD**	16	**28**		**63.64**	36.36
												
**Precision**	**92.12**	**91.18**		**Accuracy**	**0.92**		**Precision**	**90.42**	**87.50**		**Accuracy**	**0.90**
**FN**	7.88	8.82		**Kappa**	**0.75**		**FN**	9.58	12.50		**Kappa**	**0.69**
**(C) without Gender**							**(D) without Age**					
**True Class**	**Fracture**	**HIVD**		**Recall**	**FP**		**True Class**	**Fracture**	**HIVD**		**Recall**	**FP**
**Fracture**	**152**	3		**98.06**	1.94		**Fracture**	**144**	**11**		**92.90**	7.10
**HIVD**	13	**31**		**70.45**	29.55		**HIVD**	23	**21**		**47.73**	52.27
												
**Precision**	**92.12**	**91.18**		**Accuracy**	**0.92**		**Precision**	**86.23**	**65.63**		**Accuracy**	**0.83**
**FN**	7.88	8.82		**Kappa**	**0.75**		**FN**	**13.77**	**34.38**		**Kappa**	**0.47**
**(E) without Number of Pain Regions**							**(F) without Gender and Number of Pain Regions**					
**True Class**	**Fracture**	**HIVD**		**Recall**	**FP**		**True Class**	**Fracture**	**HIVD**		**Recall**	**FP**
**Fracture**	**152**	3		**98.06**	1.94		**Fracture**	**152**	**3**		**98.06**	1.94
**HIVD**	13	**31**		**70.45**	29.55		**HIVD**	**13**	**31**		**70.45**	29.55
												
**Precision**	**92.12**	**91.18**		**Accuracy**	**0.92**		**Precision**	**92.12**	**91.18**		**Accuracy**	**0.92**
**FN**	7.88	8.82		**Kappa**	**0.75**		**FN**	7.88	8.82		**Kappa**	**0.75**

**Table 6:** Comparison of classification methods with related research.

Author	Year	Task	Method	Precision	Accuracy
Koch V et al. [22]	2024	Classification of HIVDs	VNCa	0.960	0.940
Shim E et al. [23]	2022	Detecting Cervical Disc Herniation	Electron-Density Images	0.940	0.930
Mitani K et al. [26]	2023	Classification of Vertebral Body Fractures	OF Score/OF Classification	0.919	0.820
Chiari-Correia NS et al. [27]	2023	Classification of Vertebral Compression Fractures	Artificial Neural Network Model	0.935	0.933
The Presented Method	2024	Classification of HIVDs and Fractures	SVM with Key Predictors	0.981	0.920

## Data Availability

The data supporting the findings of this study are available from the corresponding authors upon reasonable request and subject to compliance with relevant requirements.
